# Lipedema in Sweden – “The LISE-study”: Quality of life and body image in women with lipedema compared to healthy controls and women with cancer

**DOI:** 10.1371/journal.pone.0355488

**Published:** 2026-08-14

**Authors:** Charlotte Goodrose-Flores, Linda Björkhem-Bergman

**Affiliations:** 1 Division of Clinical Geriatrics, Department of Neurobiology, Care Sciences and Society (NVS), Karolinska Institutet, Stockholm, Sweden; 2 Palliative Medicine, Stockholms Sjukhem, Stockholm, Sweden; University of Diyala College of Medicine, IRAQ

## Abstract

**Background:**

Women with lipedema have an abnormal accumulation of adipose tissue that leads to pain and reduced quality of life (QoL). The impact of lipedema on body image and QoL remains largely unknown. The aim of this study is to explore Health-Related QoL (HR-QoL) and body image in women with lipedema and compare it to healthy women without lipedema as well as women with advanced cancer.

**Methods:**

This comparative study included women with lipedema (n = 104), healthy women without lipedema (healthy controls) (n = 42), and an exploratory group of women with advanced cancer (n = 44). HR-QoL was assessed using RAND-36, body image concern was evaluated with the Body Shape Questionnaire (BSQ), and symptoms were assessed with Edmonton Symptom Assessment System (ESAS).

**Results:**

In total, 190 women were included. Women with lipedema had significantly lower HR-QoL compared to healthy women without lipedema across all eight dimensions (*p* < 0.001). Women with lipedema had HR-QoL scores comparable to those of women with advanced cancer in four dimensions: physical functioning, bodily pain, and emotional problems. In the general health dimension, women with lipedema had even lower scores than women with advanced cancer (p < 0.05). Body image concerns were significantly greater in the lipedema group than in healthy controls (*p* = 0.001). Women with lipedema experienced significantly more symptoms than healthy controls, including more pain, fatigue, depression, anxiety, loss of appetite, dyspnea and reduced QoL (<0.001 for all).

**Conclusion:**

Women with lipedema had as low HR-QoL as women with advanced cancer in several dimensions, significant body image concerns and high symptom burden. These findings underscore the need to develop targeted health care initiatives for these patients.

## Introduction

Lipedema is believed to be a hereditary condition characterized by the disproportionate accumulation of subcutaneous fat, primarily in the limbs, sparing the feet and hands. About 80% of affected individuals experience fat depositions in the arms [1–3]. Although lipedema was described in the 1940s it has been largely unrecognized throughout the decades and treatment options remain limited and insufficiently defined [1,4,5].

The condition is still frequently misdiagnosed as obesity or lymphedema, resulting in delays in both diagnosis and proper management within the healthcare system. Consequently, the average age at diagnosis is typically in the thirties or later, although it may already be present in children [6,7]. Obesity is one of the most common comorbidities of lipedema. However, unlike adipose tissue in obesity, lipedema-associated fat is resistant to conventional weight loss strategies [8,9]. Other frequent comorbidities include allergies, hypothyroidism, sleep disorders and depression [10]. Lipedema has been recognized by the World Health Organization since 2018 and classified in the International Classification of Disease, 11th Revision (ICD-11) under code EF02.2, which may increase awareness and recognition of the disease [6,11].

Lipedema primarily affects women, with an estimated prevalence of 11% of women worldwide [12]. The disease progressively worsens, typically first appearing during puberty due to hormonal influences. Subsequent hormonal fluctuations throughout life contribute to increased fat accumulation over time [3,11].

Lipedema is divided into four stages from an early stage with no visible skin changes, but an appearance of disproportionate fat accumulation with pearl-sized nodules, to stage four with severe fat accumulation with lymphedema. In addition, five types of lipedema differentiate how the disorder manifests with fat accumulation in the buttocks and hips (type 1), buttocks and knees (type 2), buttocks down to the ankles (type 3), arms and legs (type 4), and fat accumulation limited to the lower legs [2,3,6]. Commonly reported symptoms of lipedema are pain, swelling of the affected areas and easy bruising [6,13].

Women with lipedema often experience fat-shaming and feel perceived by others as lacking character [14]. A reduced quality of life (QoL) is commonly reported, with further declines as symptoms such as pain and heaviness become more severe [8,15]. Due to limited treatment options, women with lipedema often seek alternative therapies outside the established healthcare system, many of which lack scientific validation [13]. Dieting is one of the most commonly chosen methods of treatment [13]. However, research on dietary interventions has been largely limited to examining the benefits of a low-carbohydrate diet [16–20].

Body image is a multidimensional concept shaped by a complex interplay of individual perception, cultural influences, and societal norms [21–23]. In addition, social media engagement today plays a crucial role in people’s lives shaping body image, self-esteem and appearance-related perceptions [24,25]. This, arguably, places additional pressure on women with lipedema, serving as a constant reminder of their distinct body shape. The perception of body image among women with lipedema remains largely unresearched. Additionally, it remains unknown how body image, and QoL, differ or intersect, between women with lipedema, those without the condition, and women with advanced disease. Understanding how women with lipedema perceive their bodies in relation to other groups, as well as evaluating their QoL, is crucial for guiding future health care initiatives to better support this underserved patient population.

The aim of this study was to explore Health Related Quality of Life (HR-QoL) and Body Image in women with lipedema and compare these outcomes to healthy women without lipedema, “the LISE-study”. To further investigate how HR-QoL of women with lipedema compares with that of other disease conditions, the results was also compared with that of women with advanced cancer, obtained from a previous study [26].

## Materials and methods

### Study design and population

This was a cross-sectional survey study, Lipedema in Sweden – “The LISE-study”. Women with lipedema, 18 years and older, were recruited between May 28, 2024, and October 10, 2024, from an advocacy group focused on lymphedema/lipedema, the Swedish Edema Association. Healthy women, without lipedema, was recruited from staff members at Stockholm’s Sjukhem (Specialized Palliative Home Care and Hospice Ward) in Stockholm, Sweden, during the same study period. To put the impaired QoL associated with lipedema into perspective, we also compared QoL with those of women with advanced cancer, admitted to Specialized Palliative Home Care, obtained from a previous study [26]. These women were diagnosed with various cancer diagnoses and at different phases of palliative care, but with a life expectancy of more than three months. Data was collected between March 19, 2018, and February 2, 2020. Data available from that study included demographic data, data on HR-QoL measured with RAND-36 and self-measured health [26].

### Sample size

The sample size calculation was based on body image concern measured with the Body Shape Questionnaire (BSQ), categorized as no concern (<80), mild concern (80–110), moderate concern (111–140), and marked concern (>140) [27]. A shift toward a higher BSQ category was considered clinically relevant. With α = 0.05 and 80% power, at least 40 participants per group were required. The final sample of 104 women with lipedema and 42 healthy controls was therefore considered adequate for the primary comparison. No separate power calculation was performed for the cancer group comparison.

### Ethical Approval and consent to participate

The study was conducted in accordance with the guidelines of the Declaration of Helsinki and approved by the Swedish Ethical Review Authority, The LISE-study Dnr: 2023-07497-01, and the study with patients with advanced cancer; Dnr: 2017/2455-31. Study participants in the LISE-study provided informed consent digitally by checking a box at the beginning of the digital informed consent form. For participants in the study with cancer patients [26], written informed consent was obtained. The obtaining of the informed consents was in accordance with the approval of the Swedish Ethical Review Authority in both studies.

### Survey instrument

The study questionnaire for women with lipedema and healthy controls was anonymous and was distributed via QR code and digital link. Collected data included demographic information (age, education level, marital status, and lipedema status), as well as RAND-36, BSQ, Edmonton Symptom Assessment System (ESAS), self-measured health, weight history, and past weight loss attempts. The women with cancer completed a paper questionnaire which included, among other items, age, BMI, self-measured health, and RAND-36.

### RAND-36

RAND-36 is a validated instrument used to measure HR-QoL [28]. This questionnaire consists of 36 questions divided into eight health domains: physical function, physical health, bodily pain, general health, vitality., social functioning, emotional problems and emotional wellbeing [28,29]. The questions are identical to the SF-36 questionnaire, a validated and widely used tool for HR-QoL assessment [30]. Items were recorded according to the RAND-36 scoring instructions, with higher values indicating better health status. For each domain, recoded item scores were transformed to a 0–100 scale, where higher scores reflected more favorable HR-QoL.

### Body shape questionnaire (BSQ)

The long form of the BSQ designed by Cooper et al was used [31]. It is a validated self-reported questionnaire with demonstrated reliability to assess body shape concern [32]. The instrument consists of 34 items rated on a six-point Likert scale from 1 (“never) to 6 (“always”), yielding a total score ranging from 34 to 204. Higher scores indicate greater concern about body shape.

### Edmonton symptom assessment system (ESAS)

The Edmonton Assessment System is a validated instrument to assess symptom burden during the last 24 hours in different disease states [33,34]. Each symptom is rated on an 11-point numerical rating scale from 0 to 10, where 0 is “no suffering” and 10 is “unbearable suffering”. A higher total score indicates a greater overall symptom burden.

### Dietary intake

Study participants in the LISE-study were asked to record their food intake during the previous day, including portion sizes, when possible, categorized by time interval. Nutritional values were calculated using the DietistNet software. Furthermore, data on hunger levels at different times of the day were collected.

### Self-measured health

Self-measured health was assessed using a vertical visual analogue scale (VAS) ranging from 0% (worst possible health) to 100% (best possible health) [35].

### Statistical analysis

Descriptive statistics were used to summarize the characteristics of study participants. Differences in categorical variables between women with lipedema and healthy controls were analyzed using Fisher’s exact test when expected frequencies were below five (e.g., special diet, use of complementary medicine, and time of hunger). Continuous variables were analyzed using independent samples t-tests for age, height (cm), weight (kg), Body Mass Index (BMI), lowest weight, weight in the previous year, and health scale. Mann-Whitney test was used to analyze the differences in HR-QoL (8 dimensions) and Body Concern in women with lipedema and healthy controls and women with advanced cancer with pair-wise comparison between the groups.

In addition, an ordered logistic regression model was used to evaluate the association between body concern and women with lipedema and healthy controls, adjusting for age and BMI. Mann-Whitney U test was conducted to examine the differences in ESAS symptoms between women with lipedema and controls. In addition, a series of multiple linear regression analyses were conducted to examine the association between women with lipedema and controls and symptom burden, measured by ESAS, and adjusted for age and BMI.

To compare intake of kilocalories, carbohydrates, fat, protein and fiber between women with lipedema and healthy controls, t-test was used. Mann-Whitney U-test was conducted to compare sitting time between women with lipedema and controls. To examine the association between pain and physical activity among women with lipedema, multiple linear regression, adjusted for age and BMI, was conducted. Mann-Whitney U-test was conducted to examine the difference in self-measured health.

Statistical significance was set at p < 0.05. All analyses were conducted using Stata (version 18.0), and GraphPad Prism version 9.0 was used to generate graphs.

## Results

### Study population

In total, 104 women with lipedema and 42 healthy women without lipedema (“healthy controls”) submitted the digital questionnaire in the LISE-study. Forty-four women diagnosed with advanced cancer were included from a previous study [26]. The women with lipedema were significantly older than the healthy controls, *p* = 0.001 (mean age was 57 compared to 49 years) and significantly younger than women with advanced cancer, (mean age 57 compared to 61.5; *p* = 0.02). Women with lipedema had a significantly higher BMI than both healthy controls and women with advanced cancer (34 compared to 25 and 24; *p* < 0.001 for both). General characteristics are summarized in Table 1.

**Table 1:** Characteristics of the study population.

	Lipedema diagnosis n = 104 Mean ± SD	Healthy controls n = 42 Mean ± SD	Cancer diagnosis n = 44 Mean ± SD
Age, years	57 ± 10	49 ± 17	61.5 ± 12.7
Height, cm	166 ± 7	170 ± 7	166.6 ± 8.1
Weight, kg	94.3 ± 22.6	70 ± 11.5	66.2 ± 12.3
Body Mass Index	34 ± 8	25 ± 4.4	23.9 ± 4.1
BMI > 25	93 ± 7	10 ± 5	24 ± 2.34
Lowest weight	67 ± 13	61 ± 8	
Wt last yr	97 ± 38	69 ± 13	
Health scale	52 ± 22.4	78 ± 18	5 ± 2
Diagnosis by a health care professional	95		
Age at diagnosis	51 ± 11		
RAND Physical function	48 ± 29	90 ± 17	55 ± 26
RAND Physical health	20 ± 20	46 ± 13	17 ± 31
RAND Emotional problems	26 ± 22	45 ± 11	31 ± 42
RAND Vitality	37 ± 20	69 ± 19	41 ± 22
RAND Emotional wellbeing	68 ± 18	84 ± 13	60 ± 18
RAND Social functioning	60 ± 27	90 ± 18	47 ± 28
RAND Pain	45 ± 20	88 ± 18	53 ± 27
RAND General health	36 ± 20	72 ± 17	42 ± 17
ESAS Pain	5.2 ± 2.2	2 ± 1.5	
ESAS Fatigue	5.7 ± 2.2	2.4 ± 1.8	
ESAS Nausea	2 ± 1.8	1.4 ± 1.3	
ESAS Depression	4.2 ± 2.4	1.7 ± 1.5	
ESAS Anxiety	3.6 ± 2.6	2 ± 1.9	
ESAS Tired	5.8 ± 2.4	2.6 ± 2.10	
ESAS Appetite	3.4 ± 2.4	1.6 ± 1.3	
ESAS Shortness of breath	3.8 ± 2.7	1.5 ± 1.11	
ESAS Quality of Life	5.5 ± 2.10	2.9 ± 2.02	
Energy (kcal)/day	1533 ± 533	1958 ± 629	
Kcal/kg	17.3 ± 7.8	29 ± 2.11	
Protein (g)/day	67 ± 2.26	73 ± 2.28	
Protein (g)/kg	0.7 ± 0.3	1.10 ± 0.4	
Carbohydrates (g)(day)	137 ± 76	252 ± 333	
Fat (g)day	75 ± 32	89 ± 41	
Fiber (g)/day	16 ± 8	21 ± 9	
	n(%)	n(%)	
**Lipedema**	104(71)	43(29)	
**Education**			
Middle school	7(7)	1(2)	
High school	25(23)	3(7)	
College/university	64(62)	37(88)	
Other	8(8)	1(2)	

### Health-related quality of life measured with RAND-36

**Fig 1:**
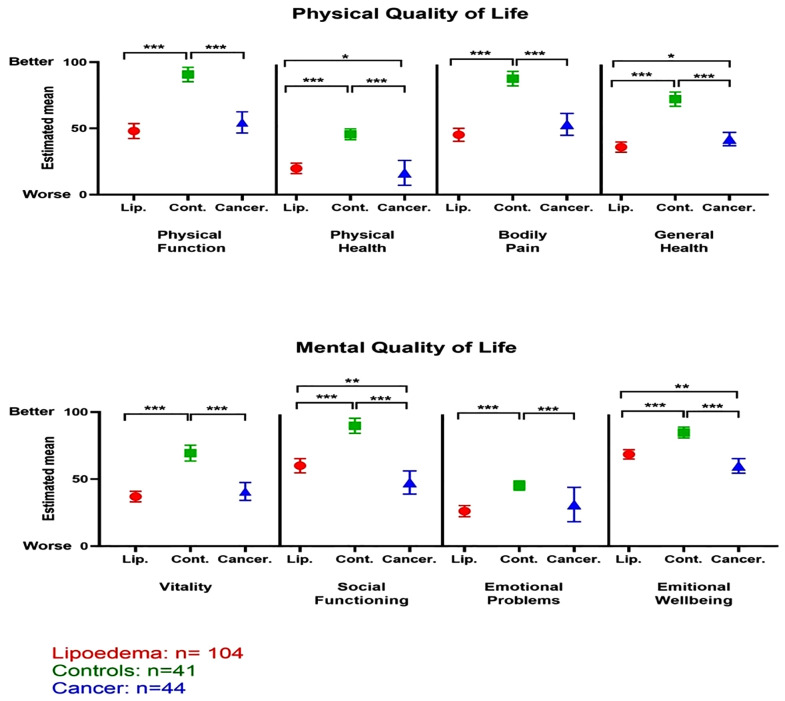
RAND-36, assessing, eight dimensions of health-related quality of life. Symbols show mean and error bars 95% CI. Statistical analysis was performed by pair-wise comparison between two groups at a time using Mann Whitney; *p < 0.05, **p < 0.01,***p < 0.001.

Women with lipedema had significantly lower HR-QoL than healthy controls in all eight dimensions: physical functioning, physical health, bodily pain, general health, vitality, social functioning, emotional problems and emotional well-being (*p* < 0.001 for all) as shown in Fig 1. Women with lipedema had HR-QoL levels comparable to those of women with advanced cancer in the dimensions physical function, bodily pain, vitality and emotional problems and even lower than those of women with advanced cancer in the general health dimension (*p* < 0.05) (Fig 1). Women with advanced cancer had lower HR-QoL than women with lipedema in the dimensions physical health (*p* < 0.05) and social functioning (p < 0.01) (Fig 1).

### Body image measured with BSQ

**Fig 2:**
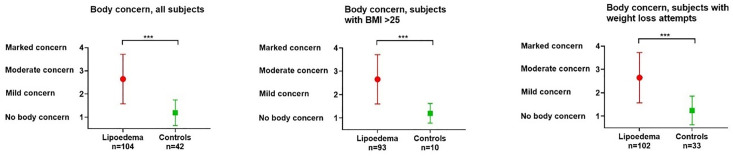
Comparing body image assessed by body shape questionnaire, in women with lipedema with healthy controls using Mann Whitney. ***p < 0.001.

There was a significant difference in body image concerns between women with lipedema and healthy controls, where women with lipedema reported a higher body image concern (Fig 2). In separate analyses, including only participants with BMI > 25 or a history of weight loss attempts to explore potential variations, the results remained unchanged (Fig 2). An ordered logistic regression model adjusted for age and BMI showed that women with lipedema had significantly higher odds of being in a higher BSQ category, indicating greater body image concern, compared with healthy controls (β = 3.41, 95% CI: 2.29–4.54; p < 0.001).

In a separate analysis including only subjects with previous weight loss attempts, women with lipedema remained significantly more likely to be in a higher BSQ category than controls, after adjusting for age and BMI β = 3.41 (95% CI: 2.29–4.54; *p* < 0.001). Additionally, in a subgroup analysis of individuals with BMI > 25, women with lipedema had significantly higher odds of being in a higher BSQ category after adjusting for age: β = 3.32 (95%CI:1.64–4.99; *p* < 0.001) compared to controls. Among women with lipedema, a higher BSQ category was significantly associated with more weight loss attempts after adjustment for age and BMI: β = 0.58 (95%CI: 0.14–1.02; *p* = 0.009). This was not observed in women without lipedema.

### Symptoms severity measured with ESAS

Women with lipedema had a significantly higher symptom burden than healthy controls: pain (*p* = 0.001), fatigue (*p* = 0.001), nausea (*p* = 0.04), depression (*p* = 0.001), anxiety (*p* = 0.001), tiredness (*p* = 0.001), appetite (*p* = 0.001), shortness of breath (*p* = 0.001) and quality of life (*p* = 0.001) (Table 1).

The results remained largely unchanged in the multiple regression analyses. (Table 2). A higher BSQ category, indicating a higher body image concern, was significantly associated with lower reported appetite in women with lipedema compared with healthy controls after adjustment for age and BMI (*p* = 0.01).

**Table 2:** A series of multiple regression analysis were conducted to compare. ESAS-symptoms between women with lipedema and healthy women without lipedema (controls), adjusted for age and BMI. Women with lipedema reported significantly higher symptom severity than controls.

ESAS-symptom	Lipedema Regression Coefficient	Age Regression Coefficient	BMI Regression Coefficient	*p*-value
Pain	2.45 ± 0.43	0.0075 ± 0.013	0.09 ± 0.023	0.001
Fatigue	2.85 ± 0.43	−0.03 ± 0.02	0.07 ± 0.02	0.001
Nausea	0.44 ± 0.40	−0.03 ± 0.01	0.03 ± 0.02	0.032
Depression	2.46 ± 0.47	−0.03 ± 0.01	0.03 ± 0.03	0.001
Anxiety	1.71 ± 0.53	−0.04 ± 0.02	0.02 ± 0.03	0.001
Tired	2.90 ± 0.50	−0.03 ± 0.02	0.05 ± 0.03	0.001
Appetite	1.8 ± 0.50	−0.03 ± 0.02	0.03 ± 0.03	0.001
Shortness of breath	1.11 ± 0.50	−0.01 ± 0.02	0.23 ± 0.03	0.001
Quality of life	2.12 ± 0.50	−0.03 ± 0.01	0.10 ± 0.03	0.001

### Dietary intake

Women with lipedema consumed significantly fewer kilocalories than healthy controls, a difference of 307 kcal, (*p* = 0.02) and significantly less carbohydrates, 52 g, (*p* < 0.01). Women with lipedema also consumed less protein, 6.7 g, less fat, 8.9 g, and less dietary fiber (3 g), than healthy controls, but the differences were not statistically significant. The reported time of greatest hunger during the day did not differ significantly between the groups.

### Physical activity

There was no significant difference in sitting time between women with lipedema and healthy controls. Among women with lipedema, there was no significant association between pain levels and weekly physical activity after adjustment for age and BMI.

### Self-measured health

We found that women with lipedema reported significantly lower self-rated health, mean 51, than healthy controls, mean 78 (*p* = 0.001), but significantly higher than that of women with advanced cancer, mean 49 (*p* = 0.001).

## Discussion

We found that women with lipedema had a significantly lower HR-QoL compared with healthy women in all eight dimensions. Their HR-QoL was comparable to that of women with advanced cancer in four dimensions; physical functioning, bodily pain, vitality and emotional problems and had even lower scores than women with advanced cancer in the general health dimension. Additionally, women with lipedema experienced significantly greater body image concerns than healthy controls. This finding remained consistent after restricting the analysis to subgroups of women with BMI > 25 or to those with previous weight loss attempts. Furthermore, a greater number of previous weight loss attempts was associated with greater body image concerns in women with lipedema. Women with lipedema also reported more severe symptoms, including pain, fatigue, depression, anxiety, tiredness, poorer appetite, and reduced quality of life, compared to healthy controls. Notably, in women with lipedema, greater body image concerns were associated with lower reported appetite compared to controls. Interestingly, women with lipedema reported lower intake of energy and carbohydrates than healthy controls.

In this study we have compared women with lipedema with women without the condition. To put the symptom burden of patients with lipedema into perspective, we also compared them with the symptom and QoL of women with advanced cancer. The comparison with women with advanced cancer should be understood as exploratory and used as a clinical reference point rather than as evidence as clinical equivalence between the two conditions.

Our finding that women with lipedema reported pain is consistent with previous literature [2,36]. However, we found that the pain levels were comparable to those reported by women with advanced cancer, which has not been previously reported. Although pain management is a standard part of cancer care, meaning that women with advanced cancer in our study may have experienced less pain than they otherwise would, our findings suggest that pain is a serious and insufficiently treated symptom in women with lipedema [37]. Impended mortality among women with advanced cancer may be considered a potential confounding factor, as it may influence HR-QoL independently of the cancer diagnosis itself. Women in palliative care may experience reduced QoL not only due to physical symptoms, but also due to existential distress related to the advanced disease. In contrast, women with lipedema have a condition that is painful but not life-threatening. Thus, the comparison between these patient groups should be interpreted with caution.

Patients with chronic edema have reported a poor body image [38,39]. To our knowledge, this is the first study to compare body image between women with lipedema and women without the condition. The results highlight the body image challenges faced by women with lipedema, who live with a body shape that differs not only from women without the condition but also from those with obesity who do not have the additional condition of lipedema.

Hunger is a physiological sensation that signals the body’s need for energy, and is primarily regulated by the hormone ghrelin [40]. In contrast, appetite is more complex and lacks reliable biological markers that link it to a specific physiological mechanism [41]. Unlike hunger, appetite is the desire to consume food, regardless of the body’s actual energy requirements or hunger state [42]. Our results reflect this complexity. The reported time of greatest hunger during the day did not differ significantly between the groups, suggesting that the physiological sensation of hunger was perceived similarly between the two groups. However, the difference in reported appetite, with controls reporting greater appetite than women with lipedema, indicates that psychological factors may be involved. Women with lipedema may be reluctant to report greater appetite, because they may perceive this as implying gluttony, potentially misrepresent them, given the challenges they face in losing weight [12].

Several studies have investigated the association between diet, weight loss, and symptoms in women with lipedema [9,16,19,20]. However, none of these studies have compared energy and macronutrient intake with that of women without the condition. Our findings underscore that women with lipedema may either have had a lower energy intake, or may have underreported their food consumption, in line with their lower reported appetite compared with controls, suggestibly to avoid being perceived as “gluttonous”. This highlights the importance of considering the complex relationship that women with lipedema may have with food intake compared with women without lipedema. Therefore, in addition to dietary changes aimed at alleviating lipedema symptoms, it may be important to specifically address potential issues to energy intake to ensure long-term success with dietary modifications.

A key strength of this study is its comparative design, which allows for a direct comparison between women with lipedema and those without the condition. This approach highlights differences in experiences related to body size and shape, providing valuable insights into the unique challenges faced by women with lipedema. We have also included women with advanced cancer as a comparison group, revealing that women with lipedema experience as low HR-QoL comparable to those of women with advanced cancer. The results underscore the profound suffering experienced by women with lipedema and is comparable to women in palliative care.

This study has several limitations. One notable limitation is the smaller sample size of the healthy control group and the group of women with advanced cancer compared with the group of women with lipedema. Although the sample size was considered adequate for the primary comparison between women with lipedema and healthy controls, no separate power calculation was performed for the comparison with the advanced cancer group. Therefore, comparisons involving women with advanced cancer should be interpreted with caution.

The fact that controls were significantly younger than the women with lipedema (mean age 49 compared to 57 years old) may also have affected the HR-QoL results, as no age-adjusted analysis was performed. Furthermore, dietary intake was self-reported and captured only consumption from the previous day, which is a limitation. Self-reported dietary data can be subject to recall bias and underreporting, especially for certain food groups. Additionally, because intake was not assessed by a dietitian**,** there may have been inaccuracies in portion-size estimation and food classification. The use of one-day dietary recall further reduces reliability, as it does not account for daily variations in dietary habits and may therefore not be representative of usual intake.

## Conclusion

In conclusion, our findings highlight the significant distress experienced by women with lipedema across multiple dimensions of HR-QoL compared to healthy controls. In fact, women with lipedema had as low HR-QoL as women with advanced cancer in several dimensions. Women with lipedema also reported greater body image concerns and a higher symptom burden than healthy controls. These results emphasize the need for targeted healthcare initiatives to address the unmet physical and psychological challenges faced by this patient population. Further research is needed to identify health care strategies that can alleviate the suffering of women with lipedema.
